# Centers of value and the quest for meaning in faith development: A measurement approach

**DOI:** 10.3389/fpsyg.2022.975160

**Published:** 2022-09-28

**Authors:** Suzanne T. Mallery, Paul Mallery

**Affiliations:** Department of Psychology, La Sierra University, Riverside, CA, United States

**Keywords:** faith development, stages of faith, meaning in life, meaning, religious styles, scale development, factor analysis, James Fowler

## Abstract

James Fowler’s model of faith development conceptualized “faith” as the quest for and maintenance of meaning oriented around centers of value which may or may not be religious or spiritual in nature. Although this model foreshadowed later work in meaning in life, substantial bodies of literature have developed in each area, almost entirely independently of the other. Integration has been hindered by measurement difficulties in faith development work. Fowler’s stages of faith development and their reformulation as Streib’s religious styles are usually measured through either a lengthy Faith Development Interview or short measures that do not assess the breadth of domains covered in the interview. These short measures are in many cases oriented around religious faith and impossible for a non-believer to answer. Embedded within the original model and the interview are aspects of faith development including perspective taking, social horizon, morality, locus of authority, form of world coherence, and symbolic function. A new Centers of Value and Quest for Meaning Scale is proposed to assess the aspects, allow non-believers to respond, tap centers of value that are not religious, and eventually address the theoretical assumption of structural wholeness across aspects. In a series of exploratory factor analyses, factors for each adult stage/style emerged for most of the aspects. This supports the potential importance of assessing the aspects and allows for more than one methodology to assess them.

## Introduction

Fowler’s (1981) model of faith development, developed from extensive interviews with 359 people, conceptualized “faith” as the quest for meaning. “Faith” in this sense is focused on questions such as for what a person spends their life; to whom someone is committed in life and death; what one’s hopes and dreams are, how one pours out their life on behalf of these goals; and with whom a person shares their most sacred hopes. These questions are parallel to the types of questions Viktor Frankl posed in his discussion of values and the will to meaning (Frankl, 1946/1992, 1992; Ryan, 2019; Wong, 2019). Following H. R. Niebuhr (Grant, 1984; Niebuhr, 1993), Fowler argued that the answers to these questions reflect the centers of value around which people base their lives (Fowler, 1981, 1986). Although “faith” is often used as a synonym to religion or spirituality (Harris et al., 2018), and almost all subsequent work using Fowler’s model has focused on religion or spirituality as the primary “center of value,” Fowler was insistent that people have meaningful centers of value that are not necessarily overtly religious or spiritual (Fowler, 1981). In this sense, Fowler’s model of faith as the quest for meaning foreshadowed and intersects with more recent work on meaning in life, but like other areas closely tied to issues of meaning (George and Park, 2016), Fowler’s model and the meaning in life literature have developed largely independently.

Fowler’s view of “faith” is not synonymous with meaning in life, but his multidimensional faith development model overlaps with and includes four key features that contribute to work on meaning in life and values. First, work on meaning in life often distinguishes between the presence of and the search for meaning in life (Steger et al., 2006, 2008). Frankl (1946/1992) also described this distinction, naming the elements “fulfillment of meaning” or “meaning in life” (presence) and the “will to meaning” (search), both of which he viewed as comprising self-transcendence (Wong and Reilly, 2017). For Frankl (1946/1992), self-transcendence always points to something other than the self. Fowler’s (1981) concept of “faith” as a “quest for meaning” is closer Frankl’s sense of “will to meaning,” as a component of self-transcendence, in that Fowler argues that meaning is derived through the ways one relates to the “centers of value” that are of ultimate importance to the person. In other words, meaning, from Fowler’s perspective, is intimately tied to what one values and how one relates to it, and Fowler stresses the importance of self-transcendent values. Fowler’s approach dovetails with Wong’s descriptions (Wong, 2013) of a spiritual approach to meaning, based in values such as compassion and serving others and encompassing personal and social responsibility and self-transcendence. Fowler defines “faith” as “people’s evolved and evolving ways of experiencing self, others and the world (as they construct them) as related to and affected by the ultimate conditions of existence (as they construct them) and of shaping their lives’ purposes and meanings, trusts and loyalties, in light of the character of being, value and power determining the ultimate conditions of existence (as grasped in their operative images–conscious and unconscious–of them” (Fowler, 1981, pp. 92–93).

Secondly, Fowler viewed faith and the quest for meaning through a developmental lens. Reker et al. (1987) found that the extent to which people perceive meaning and purpose and how much they the desire to find meaning and purpose vary across the adult lifespan. Fowler’s definition of faith as “evolved and evolving” reflects a view that both the extent and also the ways in which people find purpose and meaning develop and change qualitatively over the lifespan. The interviews on which he based his model exhibited patterns or styles of meaning making that are more or less common in various phases of life. This developmental approach to meaning is relatively rare in the meaning in life literature (for an exception see Steger et al., 2006), but has significant promise.

Thirdly, from the perspective of an ecological systems model (Bronfenbrenner, 1979), Fowler (1981) viewed this quest for meaning, or faith, as inherently relational and reflective of the individual’s ties across various levels of the ecological framework. As a result, his model is intimately concerned with the ways in which values, meaning, purpose, trust, and loyalties reflect and are played out in a web of human relationships in the context of larger social institutions and cultural systems. This emphasis parallels the relatedness and beneficence factors discussed by Martela et al. (2018). The interpersonal focus has been strengthened in Streib and Keller’s (2018) modification of the theory. In this way, Fowler echoes Frankl’s description of the “self-transcendence of human existence,” in which meaning is to be found in the world of relationships with “something, or someone other than oneself” (Frankl, 1946/1992, 1992, p. 110). As Fowler’s model predicts, Wong (1998) found both this relational component and the sense of a fair or just society to be central to people’s implicit understanding of what makes life meaningful.

Wong’s existential positive psychology 2.0 (Wong, 2019; Wong et al., 2021, in press; Arslan and Wong, 2022) argues for a paradigm shift in positive psychology that would encompass both positive and negative emotions in the search for meaning and would incorporate self-transcendence in the face of suffering as a pathway to wellbeing. As Wong (Wong, 2019; Wong et al., in press) notes, one mechanism for this is that suffering may promote a search for meaning. Fowler (1981) elaborated this most fully in his descriptions of stage 6, arguing that the penultimate form of faith development is one in which suffering and injustice faced by any member of the human community evokes a radical response of compassion and action. In this formulation, personal and social responsibility and an orientation toward care for the human community are fundamental to both Fowler’s understanding of “faith” and Arslan and Wong’s (2022) approach to existential positive psychology and meaning-making. This is a contrast to much of the work on meaning in life from the perspective of positive psychology, which has generally focused more on individual factors such as individual motivation, needs, goals, self-actualization, positive emotions, and personal fulfillment (Maslow, 1943; Baumeister, 1991; Heine et al., 2006) as well as the individual correlates of meaning in life such as personality, wellbeing, and physical and psychological health (Reker et al., 1987; Steger et al., 2008; Seligman, 2011; Steger, 2012).

Finally, extensive qualitative research with the Faith Development Interview (FDI) has yielded a rich library of diverse individual narratives that have enriched the theory and offer additional insights about what the quest for meaning looks like in the lives of people in various cultures across the globe (see, for example, Streib et al., 2009a; Streib and Hood, 2016; Tarar et al., 2017; Streib and Klein, 2018). The qualitative data derived from these narratives also may provide a fruitful avenue for the development of future hypotheses about meaning in life.

Fowler’s classic model of faith development was based in stage models of development that were current at the time the model was created. Fowler described a set of seven sequential stages in the development of faith (numbered from zero to six) ranging from early childhood to what he called “mature” faith. Some of the assumptions of the original model, especially that development occurs in linear, irreversible stages, have not been empirically supported, as will be discussed later, and as a result the model has been refined and recharacterized as Streib’s religious styles (Streib and Keller, 2018). The stages/styles present in adults in the original/current form of the model include Mythic-Literal/Instrumental Reciprocal Faith (Stage/Style 2, originally conceptualized as a childhood stage but present in some adults), Synthetic-Conventional/Mutual Faith (Stage/Style 3), Individuative-Reflective/Individuative-Systemic Faith (Stage/Style 4), Conjunctive/Dialogical Faith (Stage/Style 5), and Universalizing Faith (Stage 6, which is not included in Streib’s model). Embedded within the model are six aspects of faith development, including perspective taking, social horizon, morality, locus of authority, form of world coherence, and symbolic function. Each of these aspects takes a specific form within a specific stage/style, with more complexity with higher stages/styles. Probably because of the religious connotations of the term “faith,” the model has not received much attention outside the psychology of religion and has had minimal influence on work on meaning in life, despite significant conceptual overlap.

This model of faith development and the qualitative and quantitative instruments designed to measure it have faced a long history of criticisms and development, focused both on underlying theoretical assumptions (some of which subsequent research has demonstrated to be unfounded, as noted above) and on psychometric properties and measurement practicalities. In this paper, we propose a Centers of Value and Quest for Meaning Scale to measure Fowler’s conception of faith development in adults that addresses many of the psychometric and pragmatic measurement concerns associated with existing scales. Furthermore, this approach accepts some theoretical assumptions but treats many of them as empirical questions.

## Theoretical background

There are four key theoretical assumptions of the original faith development model reviewed in this section: That faith develops in stages, that faith has a maturational direction toward “higher” levels of faith development, that the aspects of faith development move in sync as a structural whole, and that the model has some cultural universality. As much as possible, our measurement approach does not accept these assumptions as givens but lays the foundation for testing them.

### Assumption of stages

Although Fowler based his model of faith development on Piagetian stage models of the time (especially Kohlberg’s model), technically Fowler’s model does not meet Piaget’s formal definition of “stages” of development. “Stages” by this definition are expected to occur in a consistent and invariant sequence, with a specific overall structure and integrative criteria for each stage as well as a developmental process with a construction and completion period within each stage. A more contemporary lifespan development approach would view development as multidirectional, adaptive, and involving gains and losses (Baltes et al., 1998, 2007). Indeed, Brandt has argued persuasively that Piaget would not likely have intended or expected that broad, complex concepts such as religion or faith would be explainable by a formally defined stage model (Brandt, 2019).

The initial empirical evidence from longitudinal work does not support these assumptions of invariance and irreversibility, as there is clear evidence that some individuals evidence movement toward higher stages over time and others evidence movement toward lower stages (Streib, 2013; Streib and Hood, 2016; Eufinger et al., 2019).

Another theoretical issue centers around the extent to which, as each successive “stage” of faith development becomes operational, earlier stages are abandoned or structures of that earlier stage are reformulated. In Fowler’s view, each successive stage addresses similar life issues at a new level of complexity, and he argued that stage change is triggered by the relinquishment of one’s current form of meaning-making (Fowler, 1981). Empirical research has not supported this idea of relinquishment of previous stages, as there is significant evidence that multiple stages may be apparent within the same person at any given time (Streib and Hood, 2016; Streib and Klein, 2018; Streib et al., 2020).

Streib’s Religious Styles model (Streib, 2001, 2005; Streib and Keller, 2018) addresses most of these concerns. Rather than modeling development as discrete stages, as Fowler did, Streib and Keller (2018) argue for a model in which successive overlapping waves of religious styles intersect with one another in such a way that an individual might evidence more than one style at a time. According to this model, even once a person has primarily moved away from a particular style, residual elements of that style may still be present, and the person may also demonstrate elements of styles which they do not fully embrace. This model assumes a general sequence in the emergence of styles but allows for multidirectionality of movement (reversibility) and for the possibility that the order of stage development is not invariant. Style numbers parallel stage numbers: Style 2, Instrumental-Reciprocal, parallels Stage 2, Mythic-Literal; Style 3, Mutual, parallels Stage 3, Synthetic-Conventional; Style 4, Individuative-Systemic, parallels Stage 4, Individuative-Reflective; and Style 5, Dialogical, parallels Stage 5, Conjunctive Faith.

While maintaining the possibility of developmental progression, we do not assume invariance in stages and refer to “stages/styles” to indicate that we wish to be able to measure developmental progression but are not committed to that assumption.

### Assumption of maturational direction

Another of the central assumptions of Fowler’s model is focused on the direction of maturation. Fowler argued from philosophy, theology, and the psychology of cognitive and moral development that maturation in faith should involve increased complexity of thought, increased openness to diversity, and increased commitment (1981). Researchers have elaborated on this assumption. In conjunction with the focus of the religious styles perspective on the existence of multiple styles at once in the same person, Keller (2008) applying the perspective of Mitchell (2000) argues for an alternate conception of maturity. In Fowler’s model, and to some extent in the religious styles perspective, increases in maturity consist of the use of successively more complex styles. In Keller’s view, increased maturity might be more accurately described as the flexible ability of an individual to make use of a greater range of styles as they are appropriate to the context. From this perspective, the use of a “lower” (e.g., Style 3) approach to a particular problem rather than a higher (e.g., Style 5) approach only represents a less mature faith if the person uses this style in a non-adaptive context or because they don’t have access to more “mature” faith styles. A person with “mature” faith has the option to use any of the styles that they have acquired and selects flexibly as is appropriate to the context.

Working within these theoretical traditions, our approach does examine the constructs of complexity of thought, openness to diversity, and commitment but as implied by Keller’s approach it is important to be able to assess multiple styles at once to gain a picture of the extent to which any individual is using any particular style in addressing a particular aspect of development. Where previous approaches require the assignment of either one style overall or one style per aspect, our strategy allows for a more nuanced picture of the coexistence and relative use of multiple styles within a particular aspect.

### Assumption of structural wholeness across aspects

Fowler viewed “faith” as a structural whole, in which each of the aspects would be expected to develop simultaneously and consistently (1981). In other words, the model predicts that a person’s stage assignment ought to be the same for each of the aspects. If this is the case, measurement of faith development in any one aspect ought to be sufficient to evaluate the overall stage or style of development. In practice, empirical research examining structural wholeness across aspects has been limited and equivocal, with some studies supporting the idea that the construct is unitary (Snarey, 1991; Driedger, 1997) and other more robust studies refuting this, with differences of one or two stages found across aspects in most FDIs (Streib and Klein, 2018). Unfortunately, the method by which this prediction was tested varies across studies, so the extent that structural wholeness across aspects exists is unknown. Until it is resolved, however, caution is warranted in the use of measures that assume that faith development is a unitary construct. Our approach does not assume that the aspects are structurally whole.

Religious schemata additionally contribute to conceptualizing religious styles (Streib et al., 2010). This is a more narrowly cognitive concept than either the overall idea of “faith” in Fowler’s model or religious styles (Streib, 2001, 2005; Streib and Keller, 2018). This approach raises interesting questions about the aspects, as the relation between the three proposed schemata (Truth of Text and Teaching; Fairness, Tolerance and Rationality; and Xenosophia) and Fowler’s aspects is unclear. It is likely that these schemata are correlated with some aspects but not others; additionally, some schemata may include elements from multiple aspects, and some aspects may not be related to any of the schemata.

### Assumption of cultural universality

Fowler argued that although the specific descriptions of faith stages that he proposed and the aspects observed would not necessarily be universal across cultures, the development of faith across cultures should be “broadly analogous” (Fowler, 1981, p. 298) to the stages he described. The interviews upon which the theory was based, however, included primarily monotheistic, religious people, and the term “faith” in the psychology of religion is most commonly perceived as analogous to “spirituality” or “religion” (Harris et al., 2018) rather than in the broader sense intended by Fowler (1981, 1986) and Smith (1991), which includes both theistic and non-theistic forms of existential meaning and value. Thus it is not clear how well the theory applies to non-Christian or non-Western people. Although there has been some promising work on this (Snarey, 1991; Drewek, 1996; Lee, 1999; Ok, 2006; Tarar et al., 2017; Keller et al., 2018), conclusions are only beginning to emerge to address this assumption.

## Measurement issues

One of the central obstacles to the wider use of the faith development model within psychology has been the difficulty in operationalizing it. Fowler’s faith development theory was developed based on a structured interview which then became the means of measuring it. The FDI provides rich data for narrative and content analysis and should clearly be the measure of choice for obtaining qualitative data on faith development, but although it is often used to obtain quantitative stage scores, or more recently place individuals within a typology (Streib et al., 2019), the psychometric properties of the quantitative data obtained from it are understudied and its applicability is unclear. Several questionnaire-style measures have been developed as well. In this section, we outline the FDI and briefly summarize existing questionnaire measures of faith development as they relate to the assumptions outlined above.

### The faith development interview

The FDI was originally designed to provide the exploratory data upon which the theory would be based (Fowler, 1981). For this reason, the interview initially yielded only qualitative information. Once the theory was developed, a set of coding criteria and a quantitative metric was overlaid onto the original interview, but the interview was not designed with psychometric properties in mind. Streib and Keller have addressed many of the problematic assumptions of the original theory in the current edition of the *Manual for Faith Development Research*, now titled the *Manual for the Assessment of Religious Styles in the Faith Development Interview* (Streib and Keller, 2018), but since the original publication of *Stages of Faith* (Fowler, 1981), the questions, aspect assignments, and scoring criteria on the FDI have changed significantly. For example, 15 of the 25 questions are coded under different aspects in the 2018 edition of the FDI manual from the 2004 edition (Fowler et al., 2004). This non-standard relationship between questions and aspects presents a significant threat to the reliability of the aspects as they are currently measured and to the comparability of scores across versions. Additionally, although there are enough FDIs available to do a robust analysis of the psychometric properties of the quantitative scores obtained by the interview, limited analyses have been done to date.

Despite these limitations, many researchers have put undue emphasis on the analysis of quantitative data by assigning individuals stage scores from the FDI or focusing on ratings of individual aspects and questions. A more ideal use of the significant qualitative strengths of the instrument would be to focus primarily on content and narrative analysis (Streib, 2005) either as the focus of the work (e.g., Huffaker, 2009) or to illuminate and complement quantitative findings (as demonstrated by Keller et al., 2018).

A system of scoring the FDI to yield type categorizations has recently been developed (Streib et al., 2019, 2020). Types are potentially useful heuristic categories that identify the predominant style exhibited in an interview, but as they are primarily supported conceptually, their empirical utility is promising but unclear.

A final limitation to the usefulness of the FDI is that the process of generating scores for a large enough sample for robust statistical analysis is extremely labor intensive. Attaining reliable administration and scoring of the interview requires significant training and practice, and each interview takes 12–15 h to administer and score even for trained interviewers. Thus, it is expensive and time consuming to obtain the number of participants necessary for the types of statistical analysis that are commonly used in psychology. This investment is justified in the development of the theory and in work specifically focused on faith development, and has made significant contributions to areas such as deconversion (Streib et al., 2009b) and spirituality (Streib and Hood, 2016), but it is untenable for researchers focused on other topics who wish to measure faith development as an additional variable.

### Short questionnaire measures of faith development

Several questionnaire measures of faith development are summarized here. None of them account for the aspects of faith development as detailed in Fowler’s model, and instead typically yield an overall level of development. As discussed above, this approach relies on several assumptions that may not be justified.

The most widely used short measure is the Faith Development Scale (FDS), which was designed to be an overall self-report measuring higher or lower faith development rather than assessing specific faith stages or aspects (Leak et al., 1999). It has the time advantage of being very brief (only eight forced-choice items) but has a narrow focus on religious topics and beliefs and a Christian bias compared to the FDI. In one dissertation (Pennington, 2011), scores on the FDS did not correlate with the overall score on the FDI, nor did the FDS score correlate with any aspect score. Nevertheless, the FDS has been widely used in studies of faith development (Leak, 2009; Hart et al., 2010; James et al., 2011; McDonald, 2011; Weinstein, 2011; e.g., Anderson, 2015), and a Revised Faith Development Scale (2013) frames the scale as measuring post-conventional religious reasoning, the ability to evaluate religious ideas critically and independently, that is a marker for relatively mature spiritual development. Like the original FDS, this scale focuses on specifically religious content. If one assumes structural wholeness for the stages or styles of Fowler’s model, in effect this model is using a more refined version of a concept similar to the aspect “form of logic” (Fowler et al., 2004) which was included in the FDI until the most recent edition (Streib and Keller, 2018) as a proxy for faith development as a whole. Whether structural wholeness can be assumed for Fowler’s model is an open question, and the revised version of this scale remains primarily applicable to Christians.

Two scales have been developed to measure constructs closely related to faith stages or religious styles. The Religious Schema Scale (RSS; Streib et al., 2010) measures three religious schemata that are related to religious styles; religious styles are thought of as the result of habitually using particular schemata. Originally, Truth of Text and Teaching was proposed to be associated with Stage/Style 2 (and to a lesser extent Stage/Style 3); Fairness, Tolerance, and Rationality with Stage/Style 4 (and to a lesser extent Stage/Style 5); and Xenosophia with Stage/Style 5 (Streib et al., 2010). Further empirical work (Streib and Hood, 2016) suggests that Truth of Text and Teaching is positively associated with Stage/Style 2 and negatively with Stage/Style 4; Fairness, Tolerance, and Rationality is negatively associated with Stage/Style 2; and Xenosophia is negatively associated with Stage/Style 2 and positively with Stage/Style 5. This scale has a clear structure and reliability is good; it is less clear how these schemata relate to aspects of faith development. Although the scale is not tied to any particular religion, several items do refer to religion.

Clore and Fitzgerald (2002) combined the ideas of Fowler and Bernard Lonergan to develop a measure of faith that gives primacy to cognitive processes and assesses four aspects of faith that are similar but not identical to Fowler’s stages/Streib’s styles. This measure is theistic, in that some questions focus on God. Although they did not use the same aspects as Fowler did, their initial work supports the idea that faith development may not be unitary, and measures faith on three dimensions, Common Sense Faith (parallel to Stage/Style 3), Thoughtful Faith (parallel to Stage/Style 4), and Responsible Faith (parallel to Stage/Style 5). (A fourth dimension, Transcendent Faith, was coded as the sum of Thoughtful Faith and Responsible Faith and is not discussed further in this paper.) This scale has to our knowledge been used only in a dissertation to date (Ruchgy, 2005).

Several additional short questionnaires have been developed to measure faith development (Barnes et al., 1989; Green and Hoffman, 1989; Rose, 1991; Swensen et al., 1993; Jones, 2003; Epting, 2014). None have been used widely, and the psychometric characteristics of each are limited. Most are explicitly Christian, and all assume that participants are religious.

## Scale development strategy

In response to the challenges to the assumptions in the faith development approaches described above, and in line with many of the changes made to the FDI, this project developed an alternate questionnaire measure of Fowler’s stages/Streib’s styles including the aspects of faith development that responds both to challenges to the major theoretical assumptions of Fowler’s approach and to the psychometric shortcomings and logistical problems of the qualitative and quantitative instruments previously designed to assess it. Because the FDI serves well for those wanting extensive qualitative analysis, and the FDS and RSS provide good brief measures of faith development, this project was developed to fill the gap between these approaches with an in-depth survey that measures each aspect. This kind of instrument may be particularly useful for those wishing to relate the faith development approach to meaning making and other areas where quantitative surveys are commonly used.

As discussed above, the assumption of maturational direction and abandonment of previous styles is unresolved, and until it is resolved it is unclear whether one overall score, stage, or type can reasonably represent a person’s faith development. Because of this, this scale was designed to allow stages to emerge independently of one another as unidimensional or multidimensional rather than categorizing participants into a particular stage.

Similarly, because the assumption of structural wholeness among the aspects is an open question, a scale should ideally measure aspects individually. To date, no instrument other than the FDI attempts to measure the aspects of faith development as delineated in Fowler’s theory and reconceptualized in the *Manual for the Assessment of Religious Styles in the Faith Development Interview* (Streib and Keller, 2018). These aspects are a key focus, as many have important links to concepts that have received intensive study within psychology, sociology, and religious studies, and deserve to be examined in their own right. For this reason, this scale was designed to measure each aspect that remains in the current version of the FDI (one aspect, Form of Logic, is no longer included as it was largely redundant with Piagetian models of cognitive development).

Although the data on the cultural universality of Fowler’s model is limited, a scale that is to measure centers of superordinate value should avoid explicitly religious language whenever possible and should be developed with a sample that includes people with a variety of cultural and religious backgrounds. This scale was designed such that most questions do not assume religious or spiritual centers of value (though it was impossible to avoid such questions entirely), and a significant proportion of the participants in this study were people who profess not to be religious.

Stage/Styles 3 and 4 are most commonly present in adults, but Stage/Styles 2 and 5 are also present (Streib et al., 2020). This scale is designed to assess Stages/Styles 3 through 5, and omits Stage/Style 2, because of (a) challenges developing a scale that would be clear and possible to answer for people in such a wide range of stages/styles, (b) the challenge of assessing ethnocentrism through questionnaire measures, and (c) the original conception that this stage/style is a childhood stage that people move out of during adolescence.

Finally, although typically a scale developed in an area with so much strong theory might use confirmatory factor analytic approaches, because many of the theoretical assumptions are in debate and there has been little quantitative work examining the aspects, an exploratory factor analysis approach was deemed necessary to begin this project.

## Materials and methods

### Participants

Five samples were used to measure the six aspects: separate samples for Perspective Taking, Social Horizon, Morality, and Locus of Authority, and a combined sample for the Form of World Coherence and Symbolic Function aspects. For each sample, there were two subsamples, the first composed of college students at a small Christian university in California, and the second composed of participants from the United States collected from Mechanical Turk. Sample sizes and participant characteristics are summarized in Table 1.

**TABLE 1:** Participant demographics.

	Aspect sample				
	Perspective taking	Social horizon	Morality	Locus of authority	Form of world coherence and symbolic function
**Sample**					
Total	201	204	213	249	204
College	138	42	105	108	70
mTurk	63	162	108	141	134
**Age**					
Mean	23.97	30.9	26.53	26.97	28.91
Standard deviation	9.54	11.6	9.19	10.43	11.02
**Gender**					
Women	58%	45%	61%	41%	51%
Men	37%	50%	34%	52%	45%
Other/Decline to State	5%	6%	5%	7%	4%
**Ethnicity**					
White	28%	58%	39%	41%	50%
Hispanic	21%	18%	19%	21%	15%
Asian	29%	7%	15%	15%	20%
African American/Black	6%	6%	9%	5%	6%
Multiethnic	5%	2%	6%	4%	3%
Other/Decline to State	10%	9%	11%	14%	5%
**Religion**					
Christian	76%	55%	67%	65%	62%
Atheist/Agnostic/None	10%	29%	20%	18%	23%
Other/Decline to State	14%	15%	14%	17%	14%

### Scale item selection

A database of items measuring faith development and religiosity was developed, both from existing scales and generated by the researchers. An initial pool of 291 possible items, each identified with a possible aspect, was used in two college student samples (*N* = 394 and 344, respectively) to examine possible factor structures for the Perspective Taking, Social Horizon, and Locus of Authority aspects (in the first study) and the Morality, Form of World Coherence, and Symbolic Function aspects (in the second study). Factor analyses were conducted to examine each aspect’s items, and those factor scores were correlated with other measures. These factors (Mallery and Mallery, 2015a,b) provided an initial draft for the scales for the aspects developed in this study.

To the items developed for the initial draft of the scale, additional items were added to target missing stages/styles or constructs from faith development theory. When possible, items were written using words or ideas from the coding criteria for the FDI (Fowler et al., 2004; note that these criteria are very similar to the current edition, Streib and Keller, 2018).

For each aspect, both a standard battery of other faith development and religiosity scales was administered (used across all aspects), as well as a measure of social desirability. Several aspect-specific scales were also administered to examine the validity and aid in the interpretation of each aspect. These measures are summarized in Table 2.

**TABLE 2:** Validation scales included in samples.

Aspect/Sample	Scales administered
All aspects	Faith Development Scale (Leak et al., 1999; FDS; Leak, 2003, 2008) Intentional Faith Scale (Clore and Fitzgerald, 2002) I/E-Revised (Gorsuch and McPherson, 1989) Quest (Batson and Schoenrade, 1991a,b) Religious Schema Scale (RSS; Streib et al., 2010) Marlow-Crowne Social Desirability – Reynolds Short Form A (Reynolds, 1982) General religiousness (adapted from Rowatt et al., 2009), consisting of one item assessing self-identification as religious and three assessing religious behavior.
Perspective taking	Interpersonal Reactivity Index (Davis, 1983) Narcissism Personality Index-16 (Ames et al., 2006)
Social horizon	Identification with All Humanity (McFarland et al., 2012) Openness Subscale of the International Personality Item Pool Big 5 Markers (Goldberg, 1992, 1999) Oneness Beliefs Scale (Garfield et al., 2014) Prejudice measures (adapted from Clobert et al., 2015)
Morality	Moral Foundations Questionnaire 30 (Graham et al., 2011)
Locus of authority	Right Wing Authoritarianism (Altemeyer, 1996) Social Dominance Orientation SDO6 (Pratto et al., 1994) Experiences in Close Relationships Revised (ECR-R) Questionnaire Fraley et al., 2000
Form of World Coherence and Symbolic Function	Survey of Dictionary-based Isms (SDI-46; Saucier, 2013) Epistemological Style Inventories (Wilkinson and Migotsky, 1994)

### Data collection procedures

All data was collected using online questionnaires. Participants in the college sample were students in a General Psychology class and received partial course credit for participating. Participants in the Mechanical Turk sample were paid $1.25 to $1.60 (depending on the length of the questionnaire for the aspect in which they participated). After reading an informed consent and choosing whether to continue, participants responded to the items assessing the aspect(s) of faith development being examined, presented in random order. Other scales were then presented; the order of the scales was randomized. Finally, demographics and a measure of general religiousness were collected.

### Analytic strategy

A standard set of exploratory analyses was conducted for each aspect dataset.

First, each participant’s responses were examined for each scale to see how many questions were not answered. If a participant answered at least 90% of questions from a scale, that participant’s missing data were replaced with the mean response from all respondents for that question. Participants that answered less than 90% of the questions on a scale were dropped from analyses that included that scale.

Then, several mathematical criteria for selecting the number of factors were examined, including Very Simple Structure, the Empirical Bayesian Information Criterion, the Root Mean Residual, and an examination of scree plots. These criteria all have different foci, so they were used to determine a range of reasonable numbers of factors to be extracted based on the data.

For each of these possible numbers of factors, first an Oblimin extraction was used and the highest correlations between factors were examined. If the highest correlation between any two factors was greater than or equal to 0.20, then the oblique extraction was used; if not, an orthogonal (Varimax) extraction was used.

Next, the oblique or orthogonal solutions for each of the possible number of factors extracted were examined. The primary consideration of which solution to use (and thus, the number of factors in each scale) was which solution best fit that aspect’s theoretical underpinning and coding criteria. In cases in which it was not clear which solution was the best fit theoretically, other mathematical criteria were examined as well (such as RMSEA, Tucker Lewis Index of Factoring Reliability, and the average *R*^2^ between factors and factor score estimates).

Finally, factor scores were computed for the selected factor model, and correlations between each of these factor scores and other scales were computed.

## Results

The factor analysis for each aspect is described below, along with summaries of their correlations with other validity scales. Due to space constraints, the factor loading tables and additional statistics are included in the Supplementary Materials. The results conclude with a summary of the relationship between the factors and intrinsic religiosity, extrinsic religiosity, quest, and general religiousness.

### Perspective taking aspect

Three oblique factors emerged, related to Stages/Styles 3, 4, and either 5 or the reverse of 3. Factor loadings are presented in Supplementary Table 1.

The Defended Truth factor centers around Stage/Style 3 issues (including items such as “It makes me uncomfortable to take perspectives that are very different than my own”), and the Understanding Others factor centers around Stage/Style 4 issues (such as “When trying to understand others it is most important to understand their views”). The Not Open to Family Difference factor (with items such as “I prefer to date someone who has similar beliefs about the existence of God”) is theoretically somewhat tied to Stage/Style 3 but emerged as a separate factor from Defended Truth. The reverse of this factor—Open to Family Difference—is a key element of perspective taking at higher stages. For future analyses, the reversed factor Open to Family Difference was used.

Correlations with other faith development measures (see Table 3 for summary and Supplementary Table 2 for details) were consistent with the interpretation that Defended Truth is associated with lower levels of faith stages/styles, and that Understanding Others and Open to Family Difference are associated with higher faith stages/styles. The Defended Truth and Understanding Others factors predicted the Interpersonal Reactivity Index subscales divergently (see Table 4 and Supplementary Table 3).

**TABLE 3:** Summary of significant correlations between factor scores and measures of faith development.

				Religious schema scale			Intentional faith scale		
		
Aspect	Stage/Style	Factor	FDS	ttt	ftr	xenos	Common	Thoughtful	Responsible
Perspective taking	3	Defended truth	−-	++	−	−	++		
	
	4	Understanding Others			+++	++		+++	+++
	
	5	Open to family difference	++	−-		+	−		
	
Social horizon	3	Ingroup responsibility and boundaries	−-	+++		−	+++		
	
	4	Value difference	++		+++	++		+++	++
	
	5	Close to different others	++	−	++	+++		++	++
	
Morality	3	Follow god and group	−	+++		+	+++	++	++
	
	4	Order and stability		+	++	++	++	+++	+++
	
	5	Universal values			+++	+		++	++
	
Locus of authority	3	Authority in Groups/Leaders	−	++	−		++	+	
	
	4	Authority in individuals	+		++	+		++	++
	
Form of world coherence	3	Groups and leaders	−	−			++	+	+
	
	3	Right or wrong views			−	−	−	−	−
	
	4	Consistent and appropriate beliefs			++	+	+	++	+
	
	5	Mystery		++	+	+	++	++	++
	
Symbolic function	3	Truth and symbols	−	+++		+	+++	+	++
	
	4/5	Value symbols		+	++	++	++	+++	+++
	

Supplementary Table 2 includes means, standard deviations, and sample sizes for scales, indicates significance, and includes some additional subscales. FDS, Faith Development Scale; ttt, Truth of Text and Teaching [ttt]; ftr, Fairness, Tolerance and Rationality; and xenos, Xenosophia. +/−, Positive or negative correlation greater than 0.1. ++/–, Positive or negative correlation greater than 0.3. +++/—, Positive or negative correlation greater than 0.5.

**TABLE 4:** Summary of key correlations between aspects of faith stage/styles and validation scales.

**Aspect**	**Stage/Style**	**Factor**	**Positively predicts**	**Negatively predicts**
Perspective taking	3	Defended Truth	Interpersonal Reactivity Index: Personal Distress	Interpersonal Reactivity Index: Perspective Taking, Empathic Concern
	4	Understanding Others	Narcissism Personality Index	Interpersonal Reactivity Index: Perspective Taking, Empathic Concern
	5	Open to Family Difference		Interpersonal Reactivity Index: Perspective Taking
Social Horizon	3	Ingroup Responsibility and Boundaries	Prejudice toward Muslims, Gay People, Atheists	
	4	Value Difference	Identification with All Humanity; Oneness: Spiritual, Physical (sense of oneness with the physical universe)	
	5	Close to Different Others	Identification with All Humanity	Prejudice toward Muslims, Gay People
Morality	3	Follow God and Group	Moral Foundations: Ingroup/Loyalty, Authority/Respect, and Purity/Sanctity (the foundations endorsed more by conservatives)	
	4	Order and Stability	Moral Foundations: All five foundations	
	5	Universal Values	Moral Foundations: All five foundations, but substantially higher for Harm/Care and Fairness/Responsibility (the foundations endorsed more by liberals)	
Locus of Authority	3	Authority in Groups/Leaders	Right-Wing Authoritarianism, Social Dominance Orientation; Adult Attachment: Anxiety, Avoidance	
	4	Authority in Individuals		Right-Wing Authoritarianism, Social Dominance Orientation; Adult Attachment: Avoidance
Form of World Coherence	3	Groups and Leaders	Survey of Dictionary-Based Isms: Tradition-Oriented Religiousness (focusing on organized religion); Unmitigated Self-Interest (valuing hedonism); Subjective Spirituality (focusing on mysticism and being spiritual); Epistemological Style Inventories: Naïve Realism	Survey of Dictionary-Based Isms: Inequality-Aversion (also referred to as Egalitarianism), Communal Rationalism (dealing with rational human nature and the value of social institutions)
	3	Right or Wrong Views	Survey of Dictionary-Based Isms: Unmitigated Self-Interest; Epistemological Style Inventories: Naïve Realism	Survey of Dictionary-Based Isms: Inequality-Aversion and Communal Rationalism, Subjective Spirituality
	4	Consistent and Appropriate Beliefs	Survey of Dictionary-Based Isms: Communal Rationalism; Epistemological Style Inventories: Logical Inquiry	Survey of Dictionary-Based Isms: Self-Interest; Epistemological Style Inventories: Skeptical Subjectivism
	5	Mystery	Survey of Dictionary-Based Isms: Tradition-Oriented Religiousness, Subjective Spirituality	Survey of Dictionary-Based Isms: Unmitigated Self-Interest
Symbolic Function	3	Truth and Symbols	Survey of Dictionary-Based Isms: Tradition-Oriented Religiousness, Unmitigated Self-Interest, Subjective Spirituality	Survey of Dictionary-Based Isms: Communal Rationalism, Inequality-Aversion
	4/5	Value Symbols	Survey of Dictionary-Based Isms: Tradition-Oriented Religiousness, Subjective Spirituality	Survey of Dictionary-Based Isms: Unmitigated Self-Interest

Refer to Supplementary Tables for complete correlation tables including descriptive statistics.

### Social horizon aspect

Three oblique factors emerged, associated with Styles/Stages 3, 4, and 5 (see Supplementary Table 4): Ingroup Responsibility and Boundaries (including items such as “I am most responsible to people in my religion”) was associated with Stage/Style 3, Stage/Style 4 was associated with a factor with items indicating that that participants Value Difference (e.g., “Having people with different values and principles in a group improves the group”). The Close to Different Others factor was associated with Stage/Style 5 (including questions such as, “I feel close to people with religious views very different than mine”).

Correlations with other faith development measures (see Table 3 for summary and Supplementary Table 2 for details) were consistent with this interpretation, although the distinction between Value Difference and Close to Different Others factors was not strong. The Social Horizon factors differed in their correlations between Identification with All Humanity and Oneness (see Table 4 and Supplementary Table 5), and in correlations with prejudice toward specific targets (see Table 4 and Supplementary Table 6).

### Morality aspect

Three of the correlated five factors that emerged (see Supplementary Tables 7, 8) were related to Kohlberg’s stages of moral development, and to Stage/Styles 3, 4, and 5 of faith development (summary in Table 5); other factors were less clearly related to these theoretical approaches. Factor loadings are presented in Supplementary Table 7, and correlations between factors are in Supplementary Table 8. Key findings for the Morality aspect factors are as follows:

**TABLE 5:** The relationship between the morality factors that emerged, Kohlberg’s stages, Fowler’s stages, and religious styles.

Factor	Kohlberg stage	Fowler stage	Religious style
2 Follow God and Group	3 Interpersonal Concordance	3 Synthetic-conventional	3 Mutual
1 Order and Stability	4 Law and Order	4 Individuative-Reflective	4 Individuative-Systemic
5 Universal Values	6 Universal Ethical Principle	5 Conjunctive	5 Dialogical
4 Fairness		4 Individuative-Reflective or 5 Conjunctive?	
3 Standing for Common Values

• A Follow God and Group factor (e.g., “I believe that I must obey God’s rules in order to be right with God” and “Because the values of my social or religious group are an agreement about what is right and wrong, I usually think it’s important to respect and follow those values”) was most clearly related to Stage/Style 3; some items also seem to address Stage/Style 2.
• An Order and Stability factor (e.g., “People have a duty to do things to keep the social order”) overlaps most clearly with Kohlberg’s Stage 4 (Law and Order). The Manual for Faith Development Research (Fowler et al., 2004) describes this Kohlberg stage as most closely matching Stage/Style 4, but also states that some Law and Order responses may be related to Stage 3; in that case, however, it is based on one’s group identity and membership rather than universal principles. Some of the items in this factor are related to universal principles but others can be understood as based on one’s group membership. Although these two kinds of items both loaded on this single factor, it is a reasonably good measure of Stage/Style 4 (and, by extension, the Individuative-Systemic religious style), though in some ways related to Stage/Style 3. This interpretation is supported because of the strong correlation between this factor and the Stage/Style Universal Values factor (below; *r* = 0.41) and moderate correlation between this factor and the Follow God and Group factor (*r* = 0.26).
• A Universal Values factor (e.g., “Human beings are more important than institutions”) was most clearly related to Kohlberg’s Stage 6, Universal Ethical Principle, which in turn is parallel with Stage/Style 5.
• Two other factors, Fairness (e.g., “Things are fair when each person gives and gets the same amount”) and Common Values (e.g., “When I take a stand for one value and against another, it is often because that is what most people have agreed to”) also emerged. Because these were not clearly associated with either a Kohlberg, nor a faith development Stage/Style, these may not be relevant to the faith development approach. Fairness was, however, moderately correlated with both Order and Stability (*r* = 0.41) and Universal Values (*r* = 0.33), suggesting that it may be related to Stages/Styles 4 or 5.

Correlations with other measures of faith development (see Table 3 for summary and Supplementary Table 2 for details) are consistent with this interpretation, though the theoretical distinction between Stage/Styles 4 and 5 in the extracted factors was minimally present in the pattern of correlations. Correlation of these factors with subscales from the Moral Foundations Questionnaire (see Table 4 and Supplementary Table 9) diverged depending on whether the values are more endorsed by conservatives or liberals: Follow God and group was positively related to conservative-endorsed moral foundations, Order and Stability was positively related to all the moral foundations, and Universal Values was more positively related to liberal-endorsed moral foundations than conservative-endorsed moral foundations.

### Locus of authority aspect

Two orthogonal factors emerged that were related to Stage/Style 3 and Stage/Style 4 (Factor loadings are presented in Supplementary Table 10). Authority in Groups/Leaders (Stage/Style 3) included items focusing on trusting recognized leaders or authorities, including rules and laws (e.g., “Recognized leaders are usually the best guides to knowing what is true”). Two items (out of seven) loaded on this factor that included reference to religion (e.g., “Something is worth believing if it is traditionally accepted by people in my religion”). Authority in Individuals (Stage/Style 4) items focused on the individual needing to choose the right leaders or ideas to follow (e.g., “I carefully examine claims of people who claim to be authorities to decide whether I can support them”).

The correlations between the factors and other measures of faith development (see Table 3 for summary and Supplementary Table 2 for details) were consistent with this interpretation of these factors. Authority in Groups/Leaders was positively associated with Right Wing Authoritarianism, Social Dominance Orientation, and the avoidance dimension of adult attachment; Authority in Individuals was negatively correlated with all these other measures (see Table 4 and Supplementary Table 11).

### Form of world coherence aspect

The four oblique factors that emerged (see Supplementary Tables 12, 13) were conceptually well-aligned with Stages/Styles 3, 4, and 5. The Groups and Leaders, and Right or Wrong Views factors were both related to Stage/Style 3. Groups and Leaders, focused on groups and leaders as the source of one’s beliefs (e.g., “One of the best ways to figure out my beliefs and values is to see what respected leaders in my group believe.”). Factor 3, Right or Wrong Views, included items related to binary right-or-wrong thinking associated with ingroups and outgroups (e.g., “People whose views are different from my group’s views are probably wrong.”). Because the correlation between these oblique factors was low (*r* = 0.11), it may be that these two components are not closely related to Stage/Style 3 for this aspect; in particular, Groups and Leaders may be more closely related to the Locus of Authority aspect.

The Consistent and Appropriate Beliefs factor was tied to characteristics of Stage/Style 4 and taps into finding ways to individually make sense of the world and find contextualized solutions (e.g., “I try to make my view on the world comprehensive and clear” or “It is important to me that my beliefs and views are consistent.”). Only three items have reasonably high loadings on this factor.

The final factor, Mystery, centered on several items assessing transcendence and the limitations of knowledge (e.g., “There is a lot that is true but can’t be seen or completely understood”). This understanding is associated with Stage/Style 5, or perhaps the transition from Stage 4 to Stage 5.

The largest inter-factor correlations were between the Mystery and Groups and Leaders factors (*r* = 0.31), and between the Mystery and the Consistent and Appropriate Beliefs factor (*r* = 0.33). These moderate correlations could indicate a problem with the factors or the underlying theoretical model, or that people in higher stages have maintained some characteristics of early stages while discarding the binary thinking characteristic of earlier stages; note that the correlation between Mystery and Right or Wrong Views was small but negative (*r* = –0.14).

The relations between the Groups and Leaders factor and the Consistent and Appropriate Beliefs factor with other faith development measures were consistent with the interpretation above (see Table 3 for summary and Supplementary Table 2 for details), but the correlations between the Right or Wrong Views factor and the Mystery Factor with other faith development measures were unclear suggesting the possibility that Right or Wrong Views could be a measure of Stage/Style 2 rather than Stage/Style 3, and that the Mystery Factor may be focused on a different or broader conception of finding coherence in the world than other scales. All the factors differentially predicted some or all of Saucier’s “Isms” dimensions, and all but Mystery differentially predicted Wilkinson and Migotsky’s epistemological style top-level factors (see Table 4 and Supplementary Table 14).

### Symbolic function aspect

Two orthogonal factors emerged (Supplementary Table 15) that were related to Stage/Style 3, and Stages/Styles 4 or 5. A Value Symbols factor included the items that in the preliminary scale focused on valuing symbols (e.g., “Religious symbols mean something”) and on symbols being meaningful but not literal (e.g., “Symbols represent ideas or concepts”). This factor seems to align with higher stages/styles of faith development (Stages/Styles 4 or 5). A Symbols and Truth factor included items focusing on truth (e.g., the reverse of “Many things that people believe are myths”) and having strong feelings about symbols. This factor appears to align with lower faith development Stage/Style 3, possibly with some elements of Stage/Style 2.

Correlations with other measures of faith development or schemas seem to support this interpretation, although the pattern was not clear for the Religious Styles Scale (see Table 3 for summary and Supplementary Table 2 for details). The symbolic function factors were differentially correlated with Saucier’s “Isms” dimensions, but not with the epistemological style top-level factors (see Table 4 and Supplementary Table 14).

### Relation between the aspect factors and religiosity

Across most of the stages/styles and aspects, lower stages/styles had more positive correlations with general religiousness, intrinsic religiosity, and extrinsic religiosity than higher stages/styles, that tended to be lower, near zero, or negative. Quest, on the other hand, tended to have more positive correlations with higher than with lower stages/styles. There were exceptions to that in several cases:

• For intrinsic religiosity, the Locus of Authority aspect was not related across any stage/style, and Form of World Coherence was positively correlated with all of the stage/style factors except for one of the Stage/Style 3 factors, Right or Wrong Views, that was not related.
• For extrinsic religiosity, the correlations between Extrinsic Personal and the aspect factors were parallel to the correlations between Extrinsic Social and the aspect factors for Perspective Taking, Social Horizon, and Locus of Authority. However, morality aspect factors were positively related with Extrinsic Personal religiosity, but not Extrinsic Social religiosity. There may have been a similar divergence between Extrinsic Personal and Extrinsic Social religiosity for some stages/styles of the Form of World Coherence and Symbolic Function aspects.
• The Morality and Form of World Coherence factors were uncorrelated with Quest orientation.
• The pattern of correlations between the Form of World Coherence aspect factors and religiousness was unique: Groups and Leaders (Stage/Style 3) and Mystery (Stage/Style 5) were positively correlated with general religiousness, but Right or Wrong Views (Stage/Style 4) and Consistent and Appropriate Beliefs (Stage/Style 4) were uncorrelated.

### Summary

For all aspects except for Locus of Authority and Symbolic Function, factors emerged that were linked to Stages/Styles 3, 4, and 5 of faith development. For Locus of Authority, a two-factor solution linked to Stages/Styles 3 and 4 emerged; for Symbolic Function, a factor related to Stage/Style 3 and a factor that might be related to Stages/Styles 4 and/or 5 emerged. Table 6 summaries the factors that emerged for each aspect. The complete scale is available as an online Supplementary Material.

**TABLE 6:** Summary of stage/style factors that emerged for aspects of faith development.

	Aspect					
Stage/Style	Perspective taking	Social horizon	Morality	Locus of authority	Form of world coherence	Symbolic function
3 Synthetic-Conventional/Mutual	Defended Truth	Ingroup Responsibility and Boundaries	Follow God and Group	Authority in Groups/Leaders	Groups and Leaders; Right or Wrong Views	Truth and Symbols
4 Individuative-Reflective/Individuative Systemic	Understanding Others	Value Difference	Order and Stability	Authority in Individuals	Consistent and Appropriate Beliefs	Value Symbols
5 Conjunctive/Dialogical	Open to Family Difference	Close to Different Others	Universalizing Values		Mystery	

## Scoring and reporting results from the scale

To facilitate uniformity in use of the scale, we propose two separate scoring procedures. The first, to be used by researchers comparing results across individuals or within groups of individuals, produces endorsement scores for each aspect of each style. To calculate an endorsement score for each aspect by style, researchers average responses (corrected for direction of scoring) on each item in that aspect for that style. The result will be a score of how much the participant endorses the characteristics of, for example, style 4 perspective taking on a scale from 1 to 5, with a score of 1 indicating that the aspect as expressed by in this style is very unlike the person and a score of 5 indicating that the aspect as expressed in this style is very like the person. The aspect is scored independently for each style, allowing for the possibility that the aspect may be expressed in ways characteristic of multiple styles at the same point in a person’s life.

**FIGURE 1:**
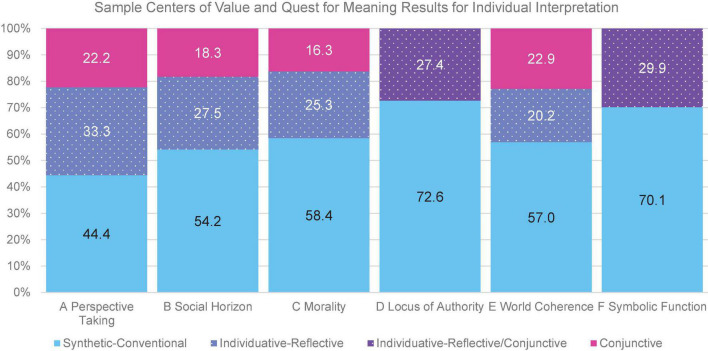
Sample stacked bar chart of simplified presentation of stages/styles by aspect for individual case interpretation.

The data from this scoring procedure may be further simplified to provide an overall profile of an individual by graphing each aspect in a stacked bar graph totaling to 100% (see Figure 1). This highlights the relative prominence of stages/styles across aspects without losing the variation between aspects and is useful for case studies or for individuals to visualize their results. In the sample figure, stage/style 3 is predominant across aspects, followed by stage/style 4 and then stage/style 5. However, there is some variance, with some aspects showing larger proportions of stage/style 4 than others, for example. In this simplification, the two World Coherence factors have been combined.

## Discussion

### Contributions to the theories of faith development and religious styles

Fowler’s original descriptions of the stages were developed in the 1970s through an analysis of interviews; it is impressive that four decades later in factor analyses of Likert-scale questions across six aspects these same core stages/styles of adult faith development would emerge (the only exception being differentiating or measuring stages/styles 4 and 5 for the Locus of Authority and Symbolic Function aspects). This approach can, with these or future data, contribute to addressing several of the theoretical and measurement issues described in the introduction.

These data support the distinction between Stages/Styles 3 (Synthetic-Conventual/Mutual Faith), 4 (Individuative Reflective/Systematic Faith), and 5 (Conjunctive/Dialogical faith). Although these stages/styles appear to be distinct, further longitudinal data is needed to identify whether they develop sequentially. Because many of these stage/style factors have small-to-moderate correlations between factors, however, the data do support a model of distinct yet overlapping styles (Streib, 2001).

This makes the assumption of maturational direction critical, and more empirical and theoretical work is needed to explore this. Despite Fowler’s broad definition of faith, his work reflects a stance in which religious forms of faith are regarded as the only truly “transcendent” centers of value (Fowler, 1981, pp. 21–23). A further elaboration of his theory has argued from ethics for xenosophia as the desired endpoint of religious development (Streib and Klein, 2018; Streib et al., 2020). In some religious traditions, however, xenosophia goes against the values of most adherents (as described by Jones, 2003). The question of potential endpoint(s) of development is to an extent a philosophical one (although a functional or evolutionary approach is certainly relevant here). Frankl’s work on meaning and self-transcendence (Frankl, 1946/1992, 1992) and Wong’s spiritual approach to meaning support the idea that a mature and meaningful endpoint might involve self-transcendent values that point beyond the individual to the importance of making moral choices that serve the good of all humanity. Fowler (1981) described stage 6 (“Universalizing faith”) as the endpoint of faith development, characterized by a radical identification with the humanity of the other which plays out through compassion and action in the face of the suffering of any member of the human family. Additionally, McFarland (2011) and McFarland et al. (2012) has argued that the ability to identify with all humanity may have been evolving throughout history.

The empirical question of whether people who are at “higher” stages have been through “lower” stages first has been addressed qualitatively, largely using self-reports based on memories of participants’ faith histories (indeed, that was Fowler’s original approach). Limited data on switching between religious types suggests that there may be a maturational direction for the stages/styles (Streib et al., 2020); cross-sectional and longitudinal work using quantitative measures such as the one developed here can help clarify this assumption of maturational direction.

The assumption of structural wholeness among aspects can also be addressed using this scale (indeed, that is the next logical step; work is underway to examine the scale using confirmatory factor analysis). Given the relatively complex nature of “faith” as defined by Fowler as encompassing moral, cognitive, religious, social, relational, and emotional components, it might be surprising to find this type of developmental, structural unity across aspects (Brandt, 2019). In both of the most recent editions of the Faith Development Manual (Fowler et al., 2004; Streib and Keller, 2018), the authors argue that the aspects should be regarded as “a heuristic model with some flexibility, rather than a rigid system with fixed boundaries” (Streib and Keller, 2018, p. 19). The extent to which aspects are tapping orthogonal characteristics or have structural unity is a question that cannot be measured using any of the current short measures of faith development. One of the strengths of this approach is that the subscales that make up the aspects have been empirically derived, and correlations between aspects in various “stages” or “styles” can be readily assessed in future work.

### The “nones” and cultural applicability

One key limitation in this scale is that samples were collected from the United States, so it is not possible to examine the assumption of cross-cultural universality. However, within this context, other paper-and-pencil measures may be more limited, as this scale has less Christian or religious language than other scales. The decades since Fowler published *Stages of Faith* have seen in the United States the rise of the “nones” (Pew Research Center, 2012) and those who label themselves as “spiritual but not religious” (Lipka and Gecewicz, 2017) as well as a range of experiences and conceptualizations of what “spiritual” means for various people (Ammerman, 2013) and what constitutes areligious spirituality (Roussiau et al., 2018). More recent research has focused on forms of transcendence that are not explicitly religious, and the evolutionary and neurological foundations for the sense of transcendence (Piedmont et al., 2009; Currier et al., 2012; Ammerman, 2013; Gorelik, 2016; Johnstone et al., 2016; Turner et al., 2018).

The FDS is especially limited in its usefulness for people who are not Christian: Of the eight items, only one item does not refer to faith or one’s Church. Across the five samples in this study, only 56% of participants completed this scale. (The last scale on the questionnaire, general religiousness, had a much higher 90% completion rate.) The revised version of the FDS (Harris and Leak, 2013) would likely have a higher completion rate as it does not require forced choices, but rating an item low could indicate either faith development or lack of church involvement. For those who did complete the FDS in this project, FDS was consistently moderately to strongly negatively correlated with the stage/style 3 factors of each aspect (see Table 3 and Supplementary Table 2). Only three of the aspects had stage/style 4 or 5 factors correlated with the FDS, however, supporting the FDS as a good overall FDS measure for people involved in a church, but one that taps into some aspects more clearly than others.

The RSS is much better for general religious audiences. No items assume a Christian perspective (and only one assumes belief in the Divine). Most of the items on the Truth of Text and Teaching subscale, though, assume that the participant is religious. Still, 74% of participants completed at least 14 of the 15 items on the RSS.

### Significance of the aspects

In addition to helping expand the applicability of questionnaire measures of faith development to individuals who are not conventionally religious, the data in this project can clarify the relationship between religious styles (as measured by the RSS) and the aspects. The Truth of Text and Teaching subscale of the RSS was strongly positively correlated with every Stage/Style 3 factor except for the Right or Wrong Views factor of the Form of World Coherence aspect. Both Fairness, Tolerance, and Rationality and Xenosophia, however, were positively related to both Stage/Styles 4 and 5 for most of the aspects (Fairness, Tolerance, and Rationality was only associated with the Understanding Others factor in the Perspective Taking aspect.) This pattern of data differs from data relating the FDI and the RSS, in which the pattern of correlations with Stage/Style 3 was less clear and Fairness, Tolerance, and Rationality was not a clear predictor of stage/style (Streib and Hood, 2016; Streib et al., 2020). Further work examining the structural wholeness of the stages/styles in the context of these measures is needed.

### “Faith” and meaning

If this scale is measuring faith in such a broad way that it is only loosely tied to religion, it is fair to ask if it is still a measure of faith development. Though constructed within the faith development approach, this scale was labeled a scale of “Meaning Making” for participants, to make it broadly accessible to those who are or are not religious. The definition of faith we have adopted here does closely parallel some definitions of meaning, including those of Frankl (1946/1992, 1992) and Wong (2013), in which both self-transcendence and personal and social responsibility to the human community are central to ideas of meaning.

This approach also shares commonalities with other work on meaning in life. Steger, for example, defined meaning in life as “the web of connections, understandings, and interpretations that help us comprehend our experience …. Meaning provides us with the sense that our lives matter, that they make sense, and that they are more than the sum of our seconds, days, and years” (Steger, 2012, p. 165). This approach to meaning in life involves three components: coherence, purpose, and existential significance (Steger, 2012; Martela and Steger, 2016; Costin and Vignoles, 2019). One of these components, coherence, involves making sense of one’s experiences, detecting patterns, and establishing a sense of predictability in the world. This may lead to an evolutionary advantage, and when disrupted, it tends to lead to a sense of distress (Martela and Steger, 2016). This dimension is parallel to the description of the Form of World Coherence aspect of Fowler’s model. Other components, purpose (which centers around motivation) and significance (which centers around value and existential meaning), are present in Fowler’s (1981) model as well and are tapped by items in the FDI, but in Fowler’s model they are described in a more communal sense than they are in the broader literature on meaning in life. For example, when the FDI asks whether people believe human life has purpose, one goal of the question is to ascertain how they navigate values, views of purpose, and authority that may conflict between self and the social environment. Thus, this approach overlaps with work on meaning of life but brings the rich and unique history of the faith development theory to the discussion.

In sum, this scale provides a useful addition for those who want to assess faith development in a more in-depth way, including assessing the aspects, than is available through other scales, but in a much more time-efficient manner than doing full FDIs. The approach taken here also allows for important assumptions of the faith development approach to be tested, particularly in relation to the aspects and structural wholeness of the stages/styles. Because the factors for each aspect varied in their patterns of correlations with a variety of relevant individual difference measures, this approach to assessing faith development may help to address the under-use of individual difference approaches in the study of religion (Streib et al., 2020).

## Data availability statement

The datasets presented in this study can be found in online repositories. The names of the repository/repositories and accession number(s) can be found below: http://doi.org/10.17605/OSF.IO/ADE4J.

## Ethics statement

The studies involving human participants were reviewed and approved by La Sierra University Institutional Review Board. The patients/participants provided their written informed consent to participate in this study.

## Author contributions

Both authors listed have made a substantial, direct, and intellectual contribution to the work, and approved it for publication.
