# Supplementary material for: Centers of value and the quest for meaning in faith development: A measurement approach
Source: Front Psychol. 2022 Sep 28;13:975160. doi: 10.3389/fpsyg.2022.975160 (PMC9554310; doi:10.3389/fpsyg.2022.975160)
Supplement: Supplementary file 1 [file Data_Sheet_1.PDF]

Supplementary Material

Table S1. Rotated Factor Matrix of Perspective Taking Aspect

	Factor		
	1 Understanding Others	2 Defended Truth	3 Not Open to Family Difference
When trying to understand others it is most important to understand their views.	.74		
I try to understand others so I can understand their views.	.67		
I think about the ways that I take other people's perspectives.	.62		
When dealing with others, I try to keep their feelings in mind.	.58		
It is very important to understand how other people feel about things.	.57		
Understanding other people's ways of looking at things gives me a better understanding of what I believe.	.56		
When trying to understand others it is most important to try to get along with them.	.56		
It is very important to understand different people's systems of thoughts.	.50		
I try to understand others so I will know what they expect of me.	.50		
Putting myself in another's shoes has helped me realize that it is OK to believe different things.	.38		
It is important for people to live in harmony.	.38		
People may seem to be different but everyone has similar wants and desires.			
It is more important to get along than to believe similar things.			
I can usually tell how people are on the inside from how they talk and act.			
I've found that some people see the world the way I do, and some people see the world in a totally different way than I do.			
It makes me uncomfortable to take perspectives that are very different than my own.		.62	
It is awkward when someone says something that is different from what I believe.		.58	
People have different illusions about life, but there is only one right way of seeing reality.		.57	
I value the different perspectives that I gain from individuals who are very different than me.	.37	-.55	
I do not value experiencing things that could threaten my religious or spiritual ideas.		.44	.32
Other people's worldviews are unique and often valuable.	.39	-.43	
Other people can reasonably disagree with my beliefs and views.		-.40	
Tolerance for other religions gets in the way of defending the truth.		.38	
I feel defensive when someone says something that is different from what I believe.		.37	
I believe it is sometimes wrong for someone to try so hard to change someone's beliefs.			
I prefer to date someone who has similar beliefs about the existence of God.			.77
It would be hard to marry someone whose beliefs are different than mine.			.74

	Factor		
	1 Understanding Others	2 Defended Truth	3 Not Open to Family Difference
I would marry someone whose beliefs are different than mine.			-.69
It does not bother me that my family members may believe something totally different than me.			-.51
People may seem to be different but in the end I've found that people see the world the way I do.		.40	-.45
A big part of why people do wrong things involves their past and current situation.			

Note: Minimum residual extraction with oblimin rotation. Factor loadings lower than .32 have been suppressed. The Defended Truth factor is negatively correlated with both Understanding Others (*r* = .-32) and Open to Family Difference (*r* = -.25). The correlation between Understanding Others and Open to Family Difference was near zero (*r* = -.03).

Table S2. Correlations Between Factor Scores and Measures of Faith Development, Religiosity, and Social Desirability.

Aspect	Stage/ Style	Factor	FDS	Religious Schema Scale ttt	ftr	xenos	Religious ID	Religious Behavior	General Religiousness
		Mean (SD)	4.10 (2.26)	16.39 (5.84)	20.91 (3.01)	17.18 (4.03)	2.75 (0.98)	0.01 (2.57)	0.05 (3.35)
		n	122	160	160	160	186	187	182
Perspective	3	Defended Truth	-.50***	.42***	-.22**	-.21**	.33***	.22**	.28***
Taking	4	Understanding Others	.12	-.01	.55***	.40***	-.01	.07	.02
	5	Open to Family Difference	.38***	-.51***	-.08	.20*	-.44***	-.48***	-.50***
		Mean (SD)	4.89 (2.36)	15.46 (6.57)	21.55 (3.14)	17.82 (4.07)	2.38 (1.14)	0.00 (2.69)	0.01 (3.56)
		n	96	137	137	137	194	190	189
Social	3	Ingroup Responsibility and Boundaries	-.63***	.56***	-.15	-.23**	.37***	.34***	.37***
Horizon	4	Value Difference	.30**	-.12	.50***	.39***	.07	.02	.04
	5	Close to Different Others	.38***	-.19*	.34***	.50***	.06	.05	.06
		Mean (SD)	4.30 (2.25)	16.74 (5.49)	20.98 (3.35)	18.02 (3.84)	2.54 (1.00)	0.02 (2.68)	0.05 (3.52)
		n	123	154	154	154	201	195	194
Morality	3	Follow God and Group	-.46***	.67***	.15	.18*	.50***	.49***	.51***
	4	Order and Stability	-.03	.19*	.47***	.38***	.10	.10	.11
	5	Universal Values	-.01	.02	.59***	.24**	-.05	.05	.03
		Fairness	-.02	.14	.38***	.34***	-.03	-.02	-.03
		Standing for Common Values	-.15	.03	-.33***	-.03	-.06	-.08	-.09
		Mean (SD)	4.87 (2.13)	15.64 (5.77)	21.73 (3.01)	18.16 (3.87)	2.53 (1.04)	0.00 (2.57)	-0.01 (3.40)
		n	141	186	186	186	228	229	225
Locus of	3	Authority in Groups/Leaders[a]	-.32***	.35***	-.19**	-.02	.28***	.24***	.28***
Authority	4	Authority in Individuals[b]	.25**	-.05	.44***	.26***	-.08	-.08	-.08
		Mean (SD)	4.61 (2.13)	15.56 (5.33)	21.25 (3.15)	17.93 (3.72)	2.53 (0.98)	0.03 (2.69)	0.11 (3.53)
		n	123	155	155	155	189	186	183
Form of	3	Groups and Leaders	-.46***	.49***	-.03	.15	.42***	.33***	.39***
World	3	Right or Wrong Views	-.09	.07	-.46***	-.41***	.04	-.06	-.02
Coherence	4	Consistent and Appropriate Beliefs[c]	-.01	.08	.34***	.18*	.08	.11	.10
	5	Mystery	.05	.31***	.25**	.22**	.30***	.27***	.29***
Symbolic	3	Truth and Symbols[d]	-.35***	.56***	.00	.18*	.56***	.54***	.57***
Function	4/5	Value Symbols[e]	.06	.29***	.42***	.35***	.31***	.31***	.32***

Table S2 (continued)

Aspect	Stage/ Style	Factor	Intentional Faith Scale: Common	Thoughtful	Responsible	Transcendent	Social Desirability
		Mean (SD)	31.11 (6.52)	41.53 (5.61)	30.05 (4.51)	71.58 (9.42)	46.10 (7.50)
		n	164	164	164	164	192
Perspective	3	Defended Truth	.31***	-.03	-.07	-.05	.06
Taking	4	Understanding Others	.14	.51***	.51***	.55***	-.01
	5	Open to Family Difference	-.29***	.00	-.01	-.01	.00
		Mean (SD)	30.34 (6.88)	42.07 (5.55)	29.73 (4.60)	71.79 (9.54)	45.23 (7.83)
		n	148	148	148	148	197
Social	3	Ingroup Responsibility and Boundaries	.50***	.10	.11	.11	.01
Horizon	4	Value Difference	.03	.54***	.41***	.51***	.03
	5	Close to Different Others	.02	.35***	.37***	.38***	.15*
		Mean (SD)	31.10 (6.99)	41.50 (6.13)	29.78 (5.10)	71.28 (10.50)	45.67 (8.10)
		n	152	152	152	152	199
Morality	3	Follow God and Group	.69***	.35***	.43***	.41***	.23***
	4	Order and Stability	.46***	.56***	.51***	.57***	.12
	5	Universal Values	.14	.38***	.38***	.41***	.06
		Fairness	.33***	.49***	.50***	.53***	.14*
		Standing for Common Values	.07	.00	-.03	-.01	.21**
		Mean (SD)	30.50 (6.31)	41.82 (5.31)	29.93 (4.88)	71.75 (9.37)	46.09 (7.91)
		n	183	183	183	183	227
Locus of	3	Authority in Groups/Leaders	.46***	.22**	.11	.18*	.26***
Authority	4	Authority in Individuals	-.03	.31***	.36***	.36***	.11
		Mean (SD)	31.42 (6.02)	41.88 (6.05)	29.56 (5.10)	71.44 (10.54)	47.15 (8.18)
		n	164	164	164	164	198
Form of	3	Groups and Leaders	.49***	.29***	.29***	.30***	.26***
World	3	Right or Wrong Views	-.19*	-.40***	-.40***	-.43***	.08
Coherence	4	Consistent and Appropriate Beliefs	.21**	.43***	.28***	.38***	-.12
	5	Mystery	.30***	.38***	.35***	.39***	.06
Symbolic	3	Truth and Symbols	.53***	.23**	.30***	.27***	.07
Function	4/5	Value Symbols	.47***	.61***	.58***	.63***	.09

Table S2 (continued)

Aspect	Stage/ Style	Factor	Intrinsic	Extrinsic Social	Extrinsic Personal	Extrinsic	Quest
		Mean (SD)	27.10 (6.06)	7.69 (2.85)	10.85 (2.61)	18.53 (4.21)	38.77 (7.93)
		n	166	166	166	166	171
Perspective	3	Defended Truth	.23**	.20**	.22**	.28***	-.36***
Taking	4	Understanding Others	.04	-.11	.11	-.01	.22**
	5	Open to Family Difference	-.60***	-.13	-.21**	-.22**	.24**
		Mean (SD)	23.61 (7.46)	5.35 (4.23)	7.90 (4.50)	13.26 (8.02)	34.21 (11.38)
		n	192	192	192	192	186
Social	3	Ingroup Responsibility and Boundaries	.37***	.34***	.34***	.37***	-.12
Horizon	4	Value Difference	-.17*	-.09	-.02	-.06	.10
	5	Close to Different Others	-.11	.13	.15*	.15*	.28***
		Mean (SD)	26.56 (5.95)	7.66 (3.17)	10.62 (3.12)	18.27 (5.05)	37.90 (7.84)
		n	156	156	156	156	164
Morality	3	Follow God and Group	.56***	.15	.52***	.42***	-.09
	4	Order and Stability	.21**	-.04	.28***	.15	.06
	5	Universal Values	.14	-.15	.22**	.04	-.06
		Fairness	.12	-.05	.37***	.20*	-.01
		Standing for Common Values	-.15	.21**	-.02	.12	.13
		Mean (SD)	26.30 (6.19)	6.99 (3.06)	10.61 (2.84)	17.60 (4.80)	39.40 (7.88)
		n	175	175	175	175	186
Locus of	3	Authority in Groups/Leaders	.10	.37***	.25***	.38***	.04
Authority	4	Authority in Individuals	-.04	-.14	.05	-.06	.29***
		Mean (SD)	25.58 (6.11)	7.93 (3.19)	10.86 (3.06)	18.79 (5.23)	38.06 (8.57)
		n	148	148	148	148	165
Form of	3	Groups and Leaders	.24**	.21**	.29***	.30***	-.10
World	3	Right or Wrong Views	.00	.15	-.16	.00	-.10
Coherence	4	Consistent and Appropriate Beliefs	.18*	-.06	.22**	.09	.00
	5	Mystery	.22**	-.13	.21*	.04	.13
Symbolic	3	Truth and Symbols	.46***	.24**	.46***	.42***	.08
Function	4/5	Value Symbols	.26**	.08	.41***	.29***	.23**

Note. FDS = Faith Development Scale; ttt = Truth of Text and Teaching [ttt]; ftr = Fairness, Tolerance and Rationality; and xenos = Xenosophia).

[a] Authorities in Groups/Leaders was moderately correlated with the Common Way from the Intentional Faith Scale (*r*(181) = .46, *p* < .001), but significantly less correlated for the Thoughtful Way (*r*(181) = .22, *p* = .003; $\bar{Z}_1^*(180) = 3.57$, *p* < .001) and Responsible Way (r(181) .11, *p* = .135; $\bar{Z}_1^*(180) = 5.09$, *p* < .001). (The test of differences between correlations is from Steiger, 1980.)

[b] Authority in Individuals was more highly correlated ($\bar{Z}_1^*(183) = 2.345$, *p* = .019) with Fairness, Tolerance, and Rationality (*r*(184) = .44, *p* < .001) than Xenosophia from the RSS (*r*(184) = .26, *p* < .001).

[c] In the Intentional Faith Scale, Consistent and Appropriate Beliefs were correlated more strongly with the Thoughtful Way (*r*(162) = .43, *p* < .001) than with the Responsible Way (*r*(162) = .28, *p* < .001, $\bar{Z}_1^*(161) = 2.95$, *p* = .003).

[d] The Truth and Symbols factor is more positively correlated ($\bar{Z}_1^* = 3.01$, *p* = .003) with the RSS' Truth of Text and Teaching factor (*r*(153) = .56, *p* < .001) than the Value Symbols factor (*r*(153) =.29, *p* < .001). The correlation between Truth and Symbols with the Common Way was higher (*r*(162) = .53, *p* < .001) than the Thoughtful (*r*(162) = .23, *p* = .003; $\bar{Z}_1^* = 5.03$, *p* < .001) or Responsible (*r*(162) = .30, *p* < .001; $\bar{Z}_1^* = 4.35$, *p* < .001) Ways.

[e] For the Intentional Faith subscales, the correlation was higher between Value Symbols and both the Thoughtful (*r*(162) = .61, *p* < .001; $\bar{Z}_1^* = 2.73$, *p* = .006) and Responsible (*r*(162) = .58, *p* < .001; $\bar{Z}_1^* = 2.31$, *p* = .021) Ways than with the Common Way (*r*(162) = .47, *p* < .001).

Table S3. Correlations between the Perspective Taking factors with the Interpersonal Reactivity Index and the Narcissism Personality Index.

		Interpersonal Reactivity Index				
Stage/ Style	Factor	Perspective Taking	Fantasy	Empathic Concern	Personal Distress	Narcissism Personality Index
	Mean	23.39	26.01	25.41	12.77	4.72
	SD	4.15	6.09	5.19	5.44	3.66
	N	187	187	187	187	170
3	Defended Truth	-.36***	-.07	-.23**	.32***	.17*
4	Understanding Others	.51***	.11	.47***	-.01	-.33***
5	Open to Family Difference	.25***	-.12	-.01	-.15*	-.04

Table S4. Rotated Factor Matrix of Social Horizon Aspect.

	Factor		
	1 Value Difference	2 Ingroup Responsibility and Boundaries	3 Close to Different Others
I value groups that have good principles and are, on the whole, useful or helpful	.62		
Having people with different values and principles in a group improves the group	.56		
I try to please my family and friends	.55		
I try to give weight to others' opinions and views as much as my own	.53		
I seek out people and ideas that are different than mine to better understand my own views	.45		
It is good to have many different ideas and viewpoints in society	.45		
My group's goals are very important to me	.45	.39	
I am most responsible to truth, whether it is found in groups or people like me or different than me	.43		
I am most responsible to people that I feel close to	.42		
All people have equal value	.39		
I am responsible to all of humanity, regardless of what groups or beliefs people have	.35		.33
Having different sources of authority makes things better			
I am most responsible to people in my religion		.69	
People are either like my religious group, or not like my religious group		.54	
I feel close to people with my same religion		.51	
It is important for me to know whether someone's beliefs are compatible with mine		.40	
I am most responsible to people who think about things like I do		.35	
I am most responsible to people in my family, ethnic, class, and/or religious groups	.33	.33	
I feel close to people with religious views very different than mine			.82
I feel close to people from different religions			.71

Note. Minimum residual extraction with oblimin rotation. Factor loadings lower than .32 have been suppressed. The Ingroup Boundaries and Responsibilities factor was positively associated with Value Difference (r = .41), and slightly negatively related to Close to Different Others (r = -.16). The correlation between Close to Different Others and Value Difference was close to zero (r = .06).

Table S5. Correlations between the Social Horizons factors with Identification with All Humanity, Openness, and Oneness scales.

				Oneness	
Stage/ Style	Factor	Ident. With All Humanity	Openness	Spiritual	Physical
	Mean	28.23	29.99	44.83	19.07
	SD	7.21	3.20	13.21	4.73
	N	190	181	177	177
3	Ingroup Responsibility and Boundaries	-.15*	.14	.10	-.21**
4	Value Difference	.31***	.04	.26***	.36***
5	Close to Different Others	.43***	-.06	.24**	.17*

Table S6. Correlations between the Social Horizons factors and prejudice towards groups.

		Prejudice						
Stage/ Style	Factor	African American	Hispanic	Asian	White	Muslim	Gay	Atheist
	Mean	15.01	15.67	16.17	16.80	12.44	12.68	14.32
	SD	4.55	4.38	4.33	3.89	5.05	4.96	5.63
	N	188	190	190	189	188	179	188
3	Ingroup Responsibility and Boundaries	.16*	.00	.13	-.04	.33***	.38***	.41***
4	Value Difference	-.25***	-.15*	-.21**	-.17*	-.12	-.19**	-.10
5	Close to Different Others	-.31***	-.16*	-.24***	-.05	-.35***	-.36***	-.14

Table S7. Rotated Factor Matrix of the Morality Aspect.

	1 Order and Stability	5 Universal Values	2 Follow God and Group	4 Fairness	3 Standing for Common Values
People have a duty to do things to keep the social order.	.69				
People have a duty to maintain order in society.	.67				
It's important to support socially agreed values, even if it means a few people lose out.	.56				
Whether something is fair or not depends on how it affects everyone involved.	.48				
The basis of moral decision-making is good character.	.45				
Sometimes it's important to limit people's rights to keep society stable.	.43				
Good laws represent good principles that are the foundation for society.	.43				
Even though a lot of rules and norms are relative, they should be followed unless they violate the rights of an individual.	.43				
It is very important that people try their best to get along without conflict.	.38				
When deciding what is right, it is important to figure out what will help the most people (or hurt the fewest people).	.37				
Laws are good because they help maintain society.	.35				
Moral decisions help people get along together.	.35				
It's wrong for people to push for their own view of what's right if their views would disrupt a good society.	.35				
People's values and opinions are relative to their cultures or groups.					
Human beings are more important than institutions.		.55			
There are some rules are good rules no matter what society they are in.		.51			
Different groups' values should be respected as long as they do not conflict with fairness and human dignity.		.49			
I help people who help me.		.47			
I usually have a rational reason for my moral decisions.		.44			
Loyalty, honesty, and sincerity are some of my best values.		.43			
When people disagree about what is right, a good solution will take both perspectives into account.		.41			
When I decide what is right, I try to think about what would happen if everyone acted as I do.		.39			

	1 Order and Stability	5 Universal Values	2 Follow God and Group	4 Fairness	3 Standing for Common Values
The rights of individuals often outweigh the rights of society.		.35			
The rights of individuals often outweigh the rights of groups.		.32			
When I take a stand for one side over another, I often think about what will help the most people.					
Society should be based on principles of justice or fairness.					
I believe that I must obey God's rules in order to be right with God.			.87		
God rewards me for what I do right, and punishes me for what I do wrong.			.69		
People have a duty to maintain order in their religious groups.			.50		
Because the values of my social or religious group are an agreement about what is right and wrong, I usually think it's important to respect and follow those values.			.42		
I feel troubled when I realize how much I participate in an evil society.			.35		
I am punished for what I do wrong.					
Things are fair when each person gives and gets the same amount.				.77	
When people want the same thing, they should be sure that everyone gets the same amount.				.61	
When there is a conflict, I try to make sure that everyone gets the same amount.				.60	
Everyone's opinions should be respected.				.36	
When I am making moral decisions, I try to step back from the agreed values of my society to consider whether how someone from a different perspective might see things.					
When I take a stand for one value and against another, it is often because that is what most people have agreed to.					.61
When I take a stand for one value and against another, it is often because I want to affirm people's dignity.					.41
People don't need to help others who don't help them.					.36
Because there any many perspectives on issues, when they conflict I have a hard time putting them together.					.34
I am rewarded for what I do right.					

Note. Minimum residual extraction with oblimin rotation. Factor loadings lower than .32 have been suppressed.

Table S8. Correlations between the Morality factors.

Style/Stage and Factor	3.	4.	5.	Fairness
3. Follow God and Group	--			
4.. Order and Stability	.26	--		
5. Universal Values	.08	.41	--	
Fairness	.13	.41	.33	--
Standing for Common Values	.13	.10	-.04	.02

Table S9. Correlations between the morality aspect factors and the Moral Foundations Questionnaire subscales

		Moral Foundations Questionnaire				
Stage/ Style	Factor	Harm/Care	Fairness/ Reciprocity	Ingroup/ Loyalty	Authority/ Respect	Purity/ Sanctity
	Mean	22.20	21.59	17.03	18.09	16.91
	SD	4.86	4.74	5.70	5.86	7.70
	N	191	191	191	191	191
3	Follow God and Group	.06	.04	.43***	.48***	.60***
4	Order and Stability	.43***	.48***	.44***	.55***	.37***
5	Universal Values	.44***	.45***	.18*	.21**	.11
	Fairness	.44***	.45***	.19**	.19**	.13
	Standing for Common Values	.05	.00	.19*	.13	.15*

Table S10. Rotated factor matrix of the Locus of Authority Aspect.

	1 Authority in Individuals	2 Authority in Group/Leaders
I carefully examine claims of people who claim to be authorities to decide whether I can support them.	.59	
I listen to multiple experts when trying to understand the world.	.56	
I want to know the reasons for guidance I am given.	.54	
A good leader should work to keep people living together without conflict.	.49	
When I follow someone, I follow because I agree with them.	.47	
I make choices after trying to view the issue from many different angles.	.43	
I think about whether rules and laws are consistent with my personal beliefs or ideas.	.39	
No single person is a completely reliable authority.	.37	
There are many good ideas, institutions, and people that I follow.	.35	
When choosing how to live, different approaches need to be evaluated in light of what is good for everyone.	.33	
The individual conscience is the best authority.		
Something is worth believing if it is traditionally accepted by people in my religion.		.62
Recognized leaders are usually the best guides to knowing what is true.		.54
When most people in my religious group believe or value something, it is probably right.		.53
Rules and laws are the proper basis for authority.		.46
I tend to trust authorities that my friends or family trust.		.43
Experts that my group values are usually worth following.		.39
It is better to follow ideas and organizations rather than individual people.		.36
I am more likely to follow good ideas or good organizations than good people.		.36
No single organization or institution is always a reliable authority.		-.34
Most of the leaders that I follow tend to be charismatic.		
I can tell if an authority is worth following if I have a good feeling about them.		

Note. Minimum residual extraction with varimax rotation. Factor loadings lower than .32 have been suppressed.

Table S11. Correlations between the Locus of Authority aspect factors and right-wing authoritarianism, social dominance orientation, and attachment.

				Attachment	
Stage/ Style	Factor	Right Wing Auth.	Social Domin. Orient.	Anxiety	Avoidance
	Mean	117.73	37.17	2.99	2.56
	SD	44.37	19.48	1.42	1.14
	N	222	229	205	205
3	Authority in Groups/Leaders	.42***	.24***	.27***	.14*
4	Authority in Individuals	-.32***	-.32***	-.09	-.17*

Table S12. Rotated Factor Matrix of the Form of World Coherence Aspect

	2 Groups and Leaders	1 Mystery	4 Consistent and Appropriate Beliefs	3 Right or Wrong Views
One of the best ways to figure out my beliefs and values is to see what respected leaders in my group believe.	.64			
One of the best ways to figure out my beliefs and values is to see what most people in my group believe.	.63			
My attitudes and beliefs are similar to my groups.	.53			
It is important that my groups don’t think too much about the values or attitudes of people who are different than us.	.52			
I know the way I look at the world is good when people or institutions I respect agree with me.	.44			
I like my groups because we share values and attitudes.	.43			
When I encounter other ways to look at the world, I try not to let them contaminate my views.	.41			
When something doesn’t make sense, it is probably not worth thinking about.	.37			
There is a lot that is true but can’t be seen or completely understood.		.73		
Many things that are true are things that we can’t see or understand.		.69		
Some things about the world can never be explained.		.63		
Sometimes things may be caused by mysterious forces that no one can understand.		.62		
Truth is complex, and doesn’t always seem to make sense.				
I try to make my view on the world comprehensive and clear.			.57	
It is important to me that my beliefs and views are consistent.			.57	
You can’t assume that a way of thinking about one problem will work for another problem.			.34	
I feel that there are some principles that can apply to everyone, even if I can’t always describe them.				
I try to understand how culture or history affects ideas in my everyday life.				
I enjoy complexity.				
When I question my religious beliefs, I don’t feel that I need to come to an immediate answer.				
People whose views are different from my group’s views are probably wrong.				.71
People have different views, but everyone’s views should be respected.				-.58
Although everyone’s views should be respected, some views are better than others.				.39

	2 Groups and Leaders	1 Mystery	4 Consistent and Appropriate Beliefs	3 Right or Wrong Views
People who think that everything is relative are wrong.				.39
I know the way I look at the world is good when it feels right to me.				

Note. Minimum residual extraction with oblique rotation. Factor loadings lower than .32 have been suppressed.

Table S13. Correlations between the Form of World Coherence factors

Style/Stage and Factor	3A.	3B.	4.
3A. Groups and Leaders	--		
3B. Right or Wrong Views	.11	--	
4. Consistent and Appropriate Beliefs	.12	-.12	--
5. Mystery	.31	-.14	0.33

Table S14. Correlations of the Form of World Coherence and Symbolic Function aspect factors with Dictionary-based "Isms" dimensions and epistemological style. *Note:* SDI-46 = Survey of Dictionary-based Isms; TR = Tradition-Oriented Religiousness; USI = Unmitigated Self-Interest; CR = Communal Rationalism; SS = Subjective Spirituality; IA = Inequality-Aversion; ESI = Epistemological Style Inventories; Naïve Real. = Naïve Realism; Logic. Inq. = Logical Inquiry; Skept. Subj. = Skeptical Subjectivism.

		SDI-46					ESI		
Stage/ Style	Factor	TR	USI	CR	SS	IA	Naïve Real.	Logic. Inq.	Skept. Subj.
	Mean	2.30	1.71	2.81	2.44	2.79	3.21	3.58	3.40
	SD	0.98	0.68	0.50	0.73	0.59	0.88	0.69	0.68
	N	182	182	182	182	182	186	186	186
Form of World Coherence Aspect									
3	Groups and Leaders	.54***	.34***	-.16*	.34***	-.30***	.40***	-.02	-.08
3	Right or Wrong Views	-.05	.30***	-.24***	-.19*	-.20**	.17*	.00	-.11
4	Consistent and Appropriate Beliefs	.06	-.25***	.25***	.04	.04	-.04	.29***	-.17*
5	Mystery	.37***	-.21**	-.01	.39***	-.04	.05	-.02	.05
Symbolic Function Aspect									
3	Truth and Symbols	.70***	.20**	-.28***	.34***	-.32***	.22**	-.18*	-.13
4/5	Value Symbols	.33***	-.24**	.12	.30***	.07	.08	.16*	.10

Table S15. Rotated Factor Matrix of the Symbolic Function Aspect

	1 Value Symbols	2 Truth and Symbols
Religious symbols mean something.	.70	
Religious symbols are useful at helping people or groups.	.65	
Symbols are important because they help people find meaning.	.63	
Symbols represent ideas or concepts.	.55	
Religious symbols mean many things.	.50	
I appreciate religious symbols even if I don't think they are real.	.48	
When thinking about important things, choosing the right words is important.	.47	
I take my religion seriously but not literally.	.34	
Many things that people believe are myths.		-.59
I feel strongly about religious symbols.		.54
Many things that people in my group believe are myths.		-.46
Religious symbols usually have one true meaning.		.37

Appendix

Centers of Value and Quest for Meaning Scale

Version 1.0

Thank you for being part of this study. Please indicate how you agree or disagree with each statement (not how you think you should answer).

Note: If you are atheist or agnostic, there may be a few items that you cannot answer, but you should still be able to answer most questions. For example, if a question asks about religious beliefs, answer the question about your beliefs about religious issues or your ideas about religious things.

Note to researcher: Items are collected using a Likert scale, anchored as follows:

1 = Strongly Disagree
2 = Disagree
3 = Completely Neutral
4 = Agree
5 = Strongly Agree

During scale development, questions have been presented in random order (by block for the paper-and-pencil version, or by item for the online version).

Items marked as "core items" are most strongly recommended by the researchers for assessing the aspects of faith development, but the scale has not been assessed using only the core items.

Core Item?	Number	Item	Aspect(s)	Stage/ Style(s)	Coding
*	1.	It makes me uncomfortable to take perspectives that are very different than my own.	Perspective Taking	3	+
*	2.	It is awkward when someone says something that is different from what I believe.	Perspective Taking	3	+
*	3.	People have different illusions about life, but there is only one right way of seeing reality.	Perspective Taking	3	+
	4.	I value the different perspectives that I gain from individuals who are very different than me.	Perspective Taking	3<br>4	-<br>+
	5.	I do not value experiencing things that could threaten my religious or spiritual ideas.	Perspective Taking	3<br>5	+<br>-
	6.	Other people's worldviews are unique and often valuable.	Perspective Taking	3<br>4	-<br>+
*	7.	Other people can reasonably disagree with my beliefs and views.	Perspective Taking	3	-
*	8.	Tolerance for other religions gets in the way of defending the truth.	Perspective Taking	3	+
*	9.	I feel defensive when someone says something that is different from what I believe.	Perspective Taking	3	+
*	10.	When trying to understand others it is most important to understand their views.	Perspective Taking	4	+

Core Item?	Number	Item	Aspect(s)	Stage/ Style(s)	Coding
*	11.	I try to understand others so I can understand their views.	Perspective Taking	4	+
*	12.	I think about the ways that I take other people's perspectives.	Perspective Taking	4	+
*	13.	When dealing with others, I try to keep their feelings in mind.	Perspective Taking	4	+
*	14.	It is very important to understand how other people feel about things.	Perspective Taking	4	+
*	15.	Understanding other people's ways of looking at things gives me a better understanding of what I believe.	Perspective Taking	4	+
*	16.	When trying to understand others it is most important to try to get along with them.	Perspective Taking	4	+
	17.	It is very important to understand different people's systems of thoughts.	Perspective Taking	4	+
	18.	I try to understand others so I will know what they expect of me.	Perspective Taking	4	+
	19.	Putting myself in another's shoes has helped me realize that it is OK to believe different things.	Perspective Taking	4	+
	20.	It is important for people to live in harmony.	Perspective Taking	4	+
*	21.	I prefer to date someone who has similar beliefs about the existence of God.	Perspective Taking	5	-
*	22.	It would be hard to marry someone whose beliefs are different than mine.	Perspective Taking	5	-
*	23.	I would marry someone whose beliefs are different than mine.	Perspective Taking	5	+
*	24.	It does not bother me that my family members may believe something totally different than me.	Perspective Taking	5	+
	25.	People may seem to be different but in the end I've found that people see the world the way I do.	Perspective Taking	5<br>3	+<br>+
*	26.	I am most responsible to people in my religion	Social Horizon	3	+
*	27.	People are either like my religious group, or not like my religious group	Social Horizon	3	+
*	28.	I feel close to people with my same religion	Social Horizon	3	+
*	29.	It is important for me to know whether someone's beliefs are compatible with mine	Social Horizon	3	+
*	30.	I am most responsible to people who think about things like I do	Social Horizon	3	+
	31.	I am most responsible to people in my family, ethnic, class, and/or religious groups	Social Horizon	3<br>4	+<br>+
*	32.	I value groups that have good principles and are, on the whole, useful or helpful	Social Horizon	4	+
*	33.	Having people with different values and principles in a group improves the group	Social Horizon	4	+
*	34.	I try to please my family and friends	Social Horizon	4	+
*	35.	I try to give weight to others' opinions and views as much as my own	Social Horizon	4	+

Core Item?	Number	Item	Aspect(s)	Stage/ Style(s)	Coding
*	36.	I seek out people and ideas that are different than mine to better understand my own views	Social Horizon	4	+
*	37.	It is good to have many different ideas and viewpoints in society	Social Horizon	4	+
	38.	My group's goals are very important to me	Social Horizon	3<br>4	+<br>+
*	39.	I am most responsible to truth, whether it is found in groups or people like me or different than me	Social Horizon	4	+
	40.	I am most responsible to people that I feel close to	Social Horizon	4	+
	41.	All people have equal value	Social Horizon	4	+
	42.	I am responsible to all of humanity, regardless of what groups or beliefs people have	Social Horizon	4<br>5	+<br>+
*	43.	I feel close to people with religious views very different than mine	Social Horizon	5	+
*	44.	I feel close to people from different religions	Social Horizon	5	+
*	45.	I believe that I must obey God's rules in order to be right with God.	Morality	3	+
*	46.	God rewards me for what I do right, and punishes me for what I do wrong.	Morality	3	+
*	47.	People have a duty to maintain order in their religious groups.	Morality	3	+
*	48.	Because the values of my social or religious group are an agreement about what is right and wrong, I usually think it's important to respect and follow those values.	Morality	3	+
*	49.	I feel troubled when I realize how much I participate in an evil society.	Morality	3	+
*	50.	People have a duty to do things to keep the social order.	Morality	4	+
*	51.	People have a duty to maintain order in society.	Morality	4	+
*	52.	It's important to support socially agreed values, even if it means a few people lose out.	Morality	4	+
*	53.	Whether something is fair or not depends on how it affects everyone involved.	Morality	4	+
*	54.	The basis of moral decision-making is good character.	Morality	4	+
*	55.	Sometimes it's important to limit people's rights to keep society stable.	Morality	4	+
*	56.	Good laws represent good principles that are the foundation for society.	Morality	4	+
*	57.	Even though a lot of rules and norms are relative, they should be followed unless they violate the rights of an individual.	Morality	4	+
	58.	It is very important that people try their best to get along without conflict.	Morality	4	+

Core Item?	Number	Item	Aspect(s)	Stage/ Style(s)	Coding
	59.	When deciding what is right, it is important to figure out what will help the most people (or hurt the fewest people).	Morality	4	+
	60.	Laws are good because they help maintain society.	Morality	4	+
	61.	Moral decisions help people get along together.	Morality	4	+
	62.	It’s wrong for people to push for their own view of what’s right if their views would disrupt a good society.	Morality	4	+
*	63.	Human beings are more important than institutions.	Morality	5	+
*	64.	There are some rules that are good rules no matter what society they are in.	Morality	5	+
*	65.	Different groups’ values should be respected as long as they do not conflict with fairness and human dignity.	Morality	5	+
*	66.	I help people who help me.	Morality	5	+
*	67.	I usually have a rational reason for my moral decisions.	Morality	5	+
*	68.	Loyalty, honesty, and sincerity are some of my best values.	Morality	5	+
*	69.	When people disagree about what is right, a good solution will take both perspectives into account.	Morality	5	+
*	70.	When I decide what is right, I try to think about what would happen if everyone acted as I do.	Morality	5	+
	71.	The rights of individuals often outweigh the rights of society.	Morality	5	+
	72.	The rights of individuals often outweigh the rights of groups.	Morality	5	+
*	73.	Something is worth believing if it is traditionally accepted by people in my religion.	Locus of Authority	3	+
*	74.	Recognized leaders are usually the best guides to knowing what is true.	Locus of Authority	3	+
*	75.	When most people in my religious group believe or value something, it is probably right.	Locus of Authority	3	+
*	76.	Rules and laws are the proper basis for authority.	Locus of Authority	3	+
*	77.	I tend to trust authorities that my friends or family trust.	Locus of Authority	3	+
*	78.	Experts that my group values are usually worth following.	Locus of Authority	3	+
	79.	It is better to follow ideas and organizations rather than individual people.	Locus of Authority	3	+
	80.	I am more likely to follow good ideas or good organizations than good people.	Locus of Authority	3	+
	81.	No single organization or institution is always a reliable authority.	Locus of Authority	3	-
*	82.	I carefully examine claims of people who claim to be authorities to decide whether I can support them.	Locus of Authority	4	+

Core Item?	Number	Item	Aspect(s)	Stage/ Style(s)	Coding
*	83.	I listen to multiple experts when trying to understand the world.	Locus of Authority	4	+
*	84.	I want to know the reasons for guidance I am given.	Locus of Authority	4	+
*	85.	A good leader should work to keep people living together without conflict.	Locus of Authority	4	+
*	86.	When I follow someone, I follow because I agree with them.	Locus of Authority	4	+
*	87.	I make choices after trying to view the issue from many different angles.	Locus of Authority	4	+
	88.	I think about whether rules and laws are consistent with my personal beliefs or ideas.	Locus of Authority	4	+
	89.	No single person is a completely reliable authority.	Locus of Authority	4	+
	90.	There are many good ideas, institutions, and people that I follow.	Locus of Authority	4	+
	91.	When choosing how to live, different approaches need to be evaluated in light of what is good for everyone.	Locus of Authority	4	+
*	92.	One of the best ways to figure out my beliefs and values is to see what respected leaders in my group believe.	Form of World Coherence	3 (Groups and Leaders)	+
*	93.	One of the best ways to figure out my beliefs and values is to see what most people in my group believe.	Form of World Coherence	3 (Groups and Leaders)	+
*	94.	My attitudes and beliefs are similar to my groups.	Form of World Coherence	3 (Groups and Leaders)	+
*	95.	It is important that my groups don't think too much about the values or attitudes of people who are different than us.	Form of World Coherence	3 (Groups and Leaders)	+
*	96.	I know the way I look at the world is good when people or institutions I respect agree with me.	Form of World Coherence	3 (Groups and Leaders)	+
*	97.	I like my groups because we share values and attitudes.	Form of World Coherence	3 (Groups and Leaders)	+
*	98.	When I encounter other ways to look at the world, I try not to let them contaminate my views.	Form of World Coherence	3 (Groups and Leaders)	+
	99.	When something doesn't make sense, it is probably not worth thinking about.	Form of World Coherence	3 (Groups and Leaders)	+
*	100.	People whose views are different from my group's views are probably wrong.	Form of World Coherence	3 (Right or Wrong)	+
*	101.	People have different views, but everyone's views should be respected.	Form of World Coherence	3 (Right or Respected)	-
*	102.	Although everyone's views should be respected, some views are better than others.	Form of World Coherence	3 (Right or Others)	+
*	103.	People who think that everything is relative are wrong.	Form of World Coherence	3 (Right or Wrong)	+

Core Item?	Number	Item	Aspect(s)	Stage/ Style(s)	Coding
*	104.	I try to make my view on the world comprehensive and clear.	Form of World Coherence	4	+
*	105.	It is important to me that my beliefs and views are consistent.	Form of World Coherence	4	+
*	106.	You can't assume that a way of thinking about one problem will work for another problem.	Form of World Coherence	4	+
*	107.	There is a lot that is true but can't be seen or completely understood.	Form of World Coherence	5	+
*	108.	Many things that are true are things that we can't see or understand.	Form of World Coherence	5	+
*	109.	Some things about the world can never be explained.	Form of World Coherence	5	+
*	110.	Sometimes things may be caused by mysterious forces that no one can understand.	Form of World Coherence	5	+
*	111.	Many things that people believe are myths.	Symbolic Function	3	-
*	112.	I feel strongly about religious symbols.	Symbolic Function	3	+
*	113.	Many things that people in my group believe are myths.	Symbolic Function	3	-
*	114.	Religious symbols usually have one true meaning.	Symbolic Function	3	+
*	115.	Religious symbols mean something.	Symbolic Function	4/5	+
*	116.	Religious symbols are useful at helping people or groups.	Symbolic Function	4/5	+
*	117.	Symbols are important because they help people find meaning.	Symbolic Function	4/5	+
*	118.	Symbols represent ideas or concepts.	Symbolic Function	4/5	+
*	119.	Religious symbols mean many things.	Symbolic Function	4/5	+
*	120.	I appreciate religious symbols even if I don't think they are real.	Symbolic Function	4/5	+
*	121.	When thinking about important things, choosing the right words is important.	Symbolic Function	4/5	+
	122.	I take my religion seriously but not literally.	Symbolic Function	4/5	+
